# High-Sensitivity Detection of the Lung Cancer Biomarker CYFRA21-1 in Serum Samples Using a Carboxyl-MoS_2_ Functional Film for SPR-Based Immunosensors

**DOI:** 10.3389/fbioe.2020.00234

**Published:** 2020-03-26

**Authors:** Nan-Fu Chiu, Hao-Tang Yang

**Affiliations:** ^1^Laboratory of Nano-photonics and Biosensors, Institute of Electro-Optical Engineering, National Taiwan Normal University, Taipei City, Taiwan; ^2^Department of Life Science, National Taiwan Normal University, Taipei City, Taiwan

**Keywords:** carboxyl-functionalized molybdenum disulfide (carboxyl-MoS_2_), surface plasmon resonance, cytokeratin 19 fragment (CYFRA21-1), immunosensors, lung cancer

## Abstract

We constructed a novel surface plasmon resonance (SPR) detection assay using carboxyl-functionalized molybdenum disulfide (carboxyl-MoS_2_) nanocomposites as a signal amplification sensing film for the ultrasensitive detection of the lung cancer-associated biomarker cytokeratin 19 fragment (CYFRA21-1). The experiment succeeded in MoS_2_ reacted with chloroacetic acid giving carboxyl-MoS_2_ as the reaction product. The additional shoulder in the C 1s and O 1s peaks of carboxyl-MoS_2_, which were increased in X-ray photoelectron spectroscopy, confirmed the presence of O–C=O groups on the surface of the carboxyl-MoS_2_. Compared to MoS_2_, the experimental results confirmed that carboxyl-modified MoS_2_ had improved low impedance and low refractive index. The carboxyl-MoS_2_-based chip had a high affinity, with an SPR angle shift enhanced by 2.6-fold and affinity binding K_*A*_ enhanced by 15-fold compared to a traditional SPR sensor. The results revealed that the carboxyl-MoS_2_-based chip had high sensitivity, specificity, and SPR signal affinity, while the CYFRA21-1 assay in spiked clinical serum showed a lower detection limit of 0.05 pg/mL and a wider quantitation range (0.05 pg/mL to 100 ng/mL). The carboxyl-MoS_2_-based chip detection value was about 10^4^ times more sensitive than the limit of detection of an enzyme-linked immunosorbent assay (ELISA) (0.60 ng/mL). The results showed that the carboxyl-MoS_2_-based chip had the potential to rapidly assay complex samples including bodily fluids, whole blood, serum, plasma, urine, and saliva in SPR-based immunosensors to diagnose diseases including cancer.

## Introduction

An early diagnosis of cancer is essential. It is the second leading cause of death worldwide, accounting for nearly one in six deaths (Ferlay et al., 2019). In 2018, there were an estimated 18.1 million new cancer cases and 9.6 million cancer deaths (Bray et al., 2018), including lung (1.76 million deaths), stomach (*n* = 782700), liver (*n* = 781600), breast (*n* = 626700), and colon (*n* = 551300) cancer (Ferlay et al., 2019). There may be no symptoms in the early or late stages of cancer, and it is often detected at the final stages of the disease. An early diagnosis of cancer can save lives and reduce medical costs, and therefore finding ways to make an early diagnosis is a very important topic. In recent years, researchers have developed various early cancer diagnostic techniques, however the traditional sensing materials commonly used at present generally lack bioaffinity characteristics. In addition, traditional biomarkers are not specific to a single cancer disease. Therefore, exploring new tumor markers and sensing materials with high specificity is an important issue in the early diagnosis of cancer.

Recently, the novel tumor marker CYFRA21-1, which is a fragment of cytokeratin-19, has been increasingly used as a tumor marker for lung cancer. CYFRA21-1 has been proven to be suitable for diagnosing non-small cell lung cancer (NSCLC) and squamous cell lung cancer (Takada et al., 1995). It is a type I keratin found in human lung cancer cells and a 40 kDa protein which is encoded by the CYFRA21-1 gene in humans. The median level in healthy individuals is 1.3 ng/mL compared to 1.5 ng/mL in individuals with benign pulmonary diseases, and a cutoff value of 3.3 ng/mL has been shown to have 95% specificity for benign lung disease (Lai et al., 1996). In 1996, Romero et al. first successfully detected lung cancer using CYFRA21-1 as a biomarker in serum samples using an ELISA with a limit of detection (LOD) of 0.60 ng/mL (Romero et al., 1996). Subsequently, Cheng S. et al. (2015) developed a field effect transistor (FET)-based biosensor that allowed for the label-free detection of CYFRA21-1 with a LOD of 1.00 ng/mL in serum samples. In addition, Staden et al. used phthalocyanine-boron dipyrromethene dye to detect CYFRA21-1, and their results showed that this method could reliably identify and determine levels of CYFRA21-1 with recoveries higher than 95% in whole blood samples (Stefan-van Staden et al., 2017). Chen et al. used an electrochemical DNA biosensor based on 3D graphene-silver nanoparticles to detect CYFRA21-1 (DNA sequence) at concentrations as low as 1.0 × 10^–14^ M in lung cancer samples (Chen et al., 2018). In addition, Zeng et al. (2018) used an electrochemical immunosensor to detect CYFRA21-1 based on 3D graphene-gold nanoparticles at a concentration ranging from 0.25 to 800 ng/mL and an LOD of 100 pg/mL in lung cancer clinical serum samples. Surface plasmon resonance (SPR) has emerged as an important highly sensitive sensing technique that does not require any labeling and allows for high-affinity detection (Kabashin et al., 2009; Sreekanth et al., 2016; Garoli et al., 2019). In our previous study, we reported a carboxyl-graphene oxide (GO-COOH)-based SPR biosensor which could detect cytokeratin 19 in 10% blood plasma at levels as low as 0.05 pg/mL and in PBS solution at levels as low as 0.001 pg/mL (Chiu et al., 2018a). In addition, Wang et al. (2016) reported an SPR biosensor based on a signal amplification strategy using antibody-quantum dot (QD) conjugates for the quantitative detection of CYFRA21-1 which had an LOD of 0.1 ng/mL in clinical samples.

The development of new sensing materials has received great attention, and especially graphene-like two-dimensional (2D) layered nanomaterials such as graphene (Singh et al., 2015; Chiu et al., 2018b), graphene oxide (GO) (Chiu and Huang, 2014; Stebunov et al., 2015; Chiu et al., 2017a), molybdenum disulfide (MoS_2_) (Lee et al., 2014; Zhang et al., 2015), and functionalized-GO (Chiu et al., 2017b) and functionalized-MoS_2_ (Chiu and Lin, 2018) nanocomposites. We successfully demonstrated that the modified carboxyl film could enhance sensing sensitivity. In addition, we previously reported the development of an aptasensor using carboxyl-GO to detect human chorionic gonadotropin (hCG) protein in clinical serum samples (Chiu et al., 2019a). Also, an immunosensor by using carboxyl-GO to detect pregnancy-associated plasma protein A2 in human plasma (Chiu et al., 2019b). The main advantages of these graphene-like nanocomposite materials are their unique functional group structures, ease of synthesis, cost-effectiveness, excellent bioaffinity, sensitivity and selectivity. Among them, MoS_2_ is regarded to be the most sensitive sensing material (Lee et al., 2014). The physicochemical properties of MoS_2_ include biocompatibility, fast electron transfer rate, excellent high electrical conductivity, quenching effect, and abundant molybdenite in the natural environment (Appel et al., 2016; Anichini et al., 2018). MoS_2_ is composed of molybdenum (Mo) and sulfur (S) in sandwich layers, and it is one of the most studied two-dimensional layered transition metal dichalcogenide (TMDC) nanomaterials. Park et al. used multilayer MoS_2_-based FET biosensors to detect prostate cancer antigen (PSA) at concentrations as low as 100 fg/mL (Park et al., 2017). In addition, Yoon et al. (2017) used a MoS_2_ nanoparticle-GO hybrid structure in an electrochemical biosensor composed of myoglobin to detect H_2_O_2_ with an LOD of 20 nM. Soni et al. (2019) used EC biosensors based on polyaniline-MoS_2_ hybrid nanostructures to detect chronic myelogenous leukemia (CML) with an LOD of 3 × 10^–18^ M, and Zhu et al. (2013) used single-layer MoS_2_-based fluorogenic nanoprobes to detect DNA with an LOD of 500 pM. In addition, Liu et al. (2017) used an MoS_2_ QD-based fluorescent biosensor to detect the breast cancer biomarker MUC1 with an LOD of 0.5 nM. However, there have been no reports on the use of MoS_2_-based biosensors to detect lung cancer biomarkers in human serum samples. In our previous study, we used a multi-layer carboxyl-functionalized molybdenum disulfide (carboxyl-MoS_2_)-based SPR biosensor to detect bovine serum albumin (BSA) protein in PBS buffer (Chiu and Lin, 2018).

However, this single-layer carboxyl-MoS_2_-based SPR biosensor has not been used for the possible interference assessment in serum testing. The goal of this study was to assess the feasibility of accurately detecting the main lung cancer biomarker CYFRA21-1 in spiked serum sample.

In this work, we successfully demonstrated that the single-layer carboxyl-MoS_2_-based SPR chip had strong interfacial interactions, preserve the advantages of the original MoS_2_ and exhibited excellent specificity, selectivity, sensitivity and affinity to detect the lung cancer biomarker CYFRA21-1 in human serum. However, the carboxyl-MoS_2_-based SPR chip had the advantages of a special glycan matrix shape, high surface area of carboxylic acid groups, and excellent bioaffinity characteristics compared to the traditional SPR (bare gold) chip. These properties suggested that the carboxyl-MoS_2_-based SPR chip would be an excellent sensing material and biosensor.

## Materials and Methods

### Synthesis of Carboxyl-MoS_2_ Nanocomposites

**FIGURE 1:**
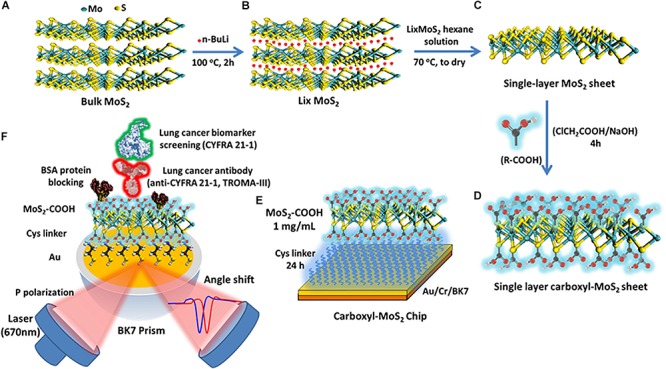
Scheme of the synthesis route of **(A)** preparation of water-dispersible bulk MoS_2_, **(B)** exfoliation of Li intercalated MoS_2_ (LixMoS_2_), **(C)** single-layer MoS_2_, **(D)** single-layer carboxyl-MoS_2_ nanocomposites, **(E)** the carboxyl-MoS_2_-based SPR chip, and **(F)** the carboxyl-MoS_2_-based SPR sensing mechanism to detect the lung cancer biomarker CYFRA21-1.

MoS_2_ powder (molybdenum IV sulfide, 234842, Sigma-Aldrich) with a purity of 99% and sheet size less than 2 μm was used (Figure 1A). Exfoliated single-layer MoS_2_ was prepared by adding 12.5 mL of anhydrous hexane solution (95%, CH_3_(CH_2_)_4_CH_3_, Sigma-Aldrich) to n-butyllithium (n-BuLi) in hexane 12.5 mL (1.6 M) to obtain a diluted 0.8 M concentration of n-BuLi in hexane in a volume of 25 mL. Next, 1 g of MoS_2_ powder was added to the prepared 25 mL of n-BuLi in hexane (0.8 M), placed in a hydrothermal synthesis reactor (Teflon), and purged with nitrogen gas (N_2_) for 5 min (Cheng Z. et al., 2015). The hydrothermal synthesis reactor was then fastened, bolted and heated to 100°C (hot plate) for 2 h as shown in Figure 1B. The prepared LixMoS_2_ was separated by high-speed centrifugation (25,000 rpm) and washed several times with an anhydrous hexane solution (95%). After centrifugation, the LixMoS_2_ anhydrous hexane solution was poured into a Teflon container and heated to 70°C to dry (to remove solution). Finally, deionized water was added to yield a single-layer MoS_2_ solution with a concentration of 1 mg/mL and a volume of 15 mL. The obtained MoS_2_ sheet could easily be prepared as a single-layer structure as shown in Figure 1C. After 1.2 g of sodium hydroxide (NaOH) and 1.0 g of chloroacetic acid (ClCH_2_COOH) were added to the single-layer MoS_2_ solution, it was shaken using ultrasound for 4 h. After homogeneous mixing, centrifugation was performed several times and the supernatant was replaced with deionized water. Ultrasonic shock waves (350 W, 60°C) were used to create vacancies in the surface of the MoS_2_ sheets, which would aid the carboxyl-functionalized modification mechanism by allowing chlorine atoms to occupy the sulfur vacancies (Presolski and Pumera, 2016). A chloroacetic acid-modified single-layer carboxyl-MoS_2_ sheet was prepared as shown in Figure 1D.

### Preparation of SPR Immunosensors and Production Program

The sensing chip was composed of a 47-nm thick gold (Au) film and a 2-nm thick chromium (Cr) thin film coating on a BK7 substrate (175 μm cover glass) created by thermal evaporation. We used the surface functionality of self-assembled monolayers (SAM) of 5 mM cystamine (Cys, cystamine dihydrochloride 96%) amino-terminated alkanethiol for 24 h to modify the chip surface, and carboxyl-MoS_2_ sheets at a concentration of 1 mg/mL to capture the –NH_2_ terminus of Cys to form chemical covalent bonds. The preparation of the carboxyl-MoS_2_-based SPR chip is shown in Figure 1E.

To immobilize antibodies, we first activated the interface of the carboxyl-MoS_2_-based chip using 0.4 mM N-ethyl-3-(3-dimethylaminopropyl) carbodiimide (EDC)/0.1 mM N-hydroxysuccinimide (NHS) at a 1:1 ratio in deionized water. For the antibody immobilization step, we used recombinant anti-CYFRA21-1 rat immunoglobulin (Ig) monoclonal antibodies (20 μg/mL) with a molecular weight of approximately 150 kDa (TROMA-III, Developmental Studies Hybridoma Bank, University of Iowa, United States) which were conjugated by direct covalent bonding onto –COOH terminals.

Anti-CYFRA21-1 proteins that were not covalently bonded on the carboxyl-MoS_2_ surface were removed using a regeneration buffer (NaOH 50 mM, pH 12.0). The blocking buffer was used at a high concentration of 500 μg/mL BSA by diluting a solution of 1× PBS to block the –COOH terminus of the remaining unbound anti-CYFRA21-1 protein on the carboxyl-MoS_2_ surface to reduce non-specific binding. Fifty mM NaCl was then used to remove excess and unbound BSA proteins on the carboxyl-MoS_2_ surface. Finally, 1 M ethanolamine (EA) solution was used to remove the remaining activated carboxylic groups on the carboxyl-MoS_2_ surface to prevent their activity and reduce the absorption of non-specific molecules on the biosensor. The material preparation and anti-CYFRA21-1 protein immobilization of the biosensor were performed using a BI-SPR 3000 dual channel instrument (Biosensing Instrument Inc., Tempe, AZ, United States). Subsequently, the antigen CYFRA21-1 molecular detection and analysis could be performed. All of the sample injections used a volume of 200 μL and flow rate of 60 μL/min at room temperature. The fabrication and detection procedures of the carboxyl-MoS_2_-based SPR immunosensor are shown in Figure 1F. The SPR instrument was equipped with a polydimethylsiloxane (PDMS) two-channel flow cell system in a channel volume of 1 μL with an inner diameter of 0.2 mm. The flow rate was set to 30–100 μL/min to observe interactions between the molecules. All data analyses of kinetic binding were performed using an analysis module in BI-DirectFlow^TM^ (Biosensing Instrument Inc., Tempe, AZ, United States). In addition, preparation of traditional SPR chip is shown in Supplementary Material.

### Considerations Regarding Real-Time Immunoassays and Preparation of Serum Samples

The aim of this study was to detect the antigen CYFRA21-1 human protein, which has a molecular weight of approximately 44 kDa (Pro-350, ProSpec-Tany TechnoGene Ltd., Israel). The CYFRA21-1 human recombinant produced in Escherichia coli (E. coli) is a single, non-glycosylated polypeptide chain. As we wished to explore potential applications involving assays of complex serum fluids, we used normal serum (non-lung cancer) from a 23-year-old female as the complex serum fluid. The CYFRA21-1 protein concentration in this serum sample was determined by ELISA (Biochrom EZ Read 400, Cambridge, United Kingdom) to be 0.2 ng/mL (ELISA kit detection range: 62.5–4000 pg/mL). The normal female serum sample was provided by Mackay Memorial Hospital (Taipei, Taiwan). All experiments were performed in compliance with the relevant laws and institutional guidelines, and the work was approved by the Institutional Review Board (IRB) of Mackay Hospital for Human Clinical Trials (Permit Number: 15MMHIS020 and 15MMHIS115).

In spiked serum experiments, the PBS-based running buffer consisted of 50 mM NaCl (10 mL), 0.05% Tween-20 (30 μL), and 1 mg/mL BSA (500 μL) mixed in 100 mL 1× PBS (this PBS/BSA/Tween-20/NaCl running buffer was abbreviated as PBS_(__*BTN*__)_). The purpose of adding NaCl, Tween-20 and BSA was to remove non-specifically adsorbed. At the same time, NaCl and Tween-20 could reduce destruction of the binding of sample proteins and antibodies on the surface of the chip. The serum-based running buffer (SRB) consisted of serum (0.1 mL), and 50 mM NaCl (5.0 mL) mixed in 10 mL PBS_(BTN)_ buffer. We used the running buffer to dilute CYFRA21-1 (Pro-350) protein to different concentrations of 0 pg/mL, 0.1 pg/mL, 1.0 pg/mL, 10 pg/mL, 100 pg/mL, 1.0 ng/mL, 10 ng/mL, 100 ng/mL, and 200 ng/mL. We called these the “initial samples.” We then mixed the initial samples with complex serum fluid at a ratio of 1:1 in a total volume of 200 μL within a concentration ranging from 0.05 pg/mL–100 ng/mL. We called this “spiked assay sample solutions.” All assay experiments were conducted at 23°C ± 2°C using a sample injection volume of 200 μL and a flow rate of 60 μL/min in the BI-SPR 3000 dual channel instrument. All assay results were repeated using the same chip detection method and various sensing channel paths.

### Preparation Procedure of Evaluated Specificity Tests

In the evaluated specificity test, we used different non-specific biomarkers at concentrations of 200 ng/mL each and a volume of 200 μL including cancer antigen 19-9 (CA-199, product number: MBS173342, MyBioSource Inc., United States), hCG (product number: 1297001, Sigma-Aldrich, United States), pregnancy-associated plasma protein A recombinant protein (PAPP-A, product number: MBS2011936, MyBioSource Inc., United States), pregnancy-associated plasma protein A2 recombinant protein (PAPP-A2, product number: MBS2011332, MyBioSource Inc., United States), and human serum albumin (HSA, ≥98%, product number: SRP618, Sigma-Aldrich, United States) proteins. In addition, in the evaluated specificity experiments, we used the six different biomarkers (CA-199, hCG, PAPP-A, PAPP-A2, HSA, and CYFRA21-1) at concentrations of 200 ng/mL each mixed with S5 (6.25% serum) at a ratio of 1:1 in a total volume of 200 μL. The evaluated specificity tests were conducted at room temperature (23°C), using a sample injection volume of 200 μL and a flow rate of 60 μL/min.

## Results and Discussion

### Analysis and Characterization of the Carboxyl-MoS_2_ Nanocomposite

**FIGURE 2:**
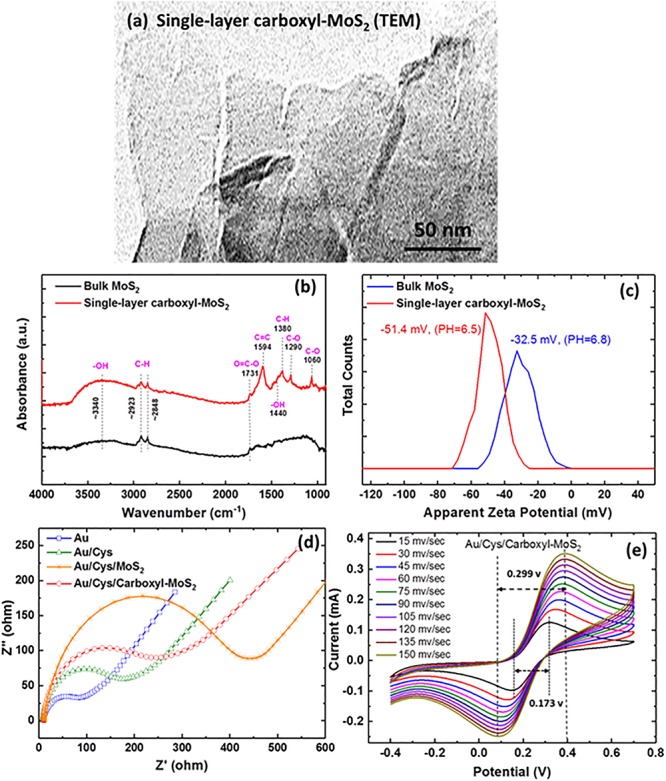
**(a)** TEM images of the single-layer carboxyl-MoS_2_ nanocomposite. **(b)** FTIR spectra of bulk MoS_2_ and single-layer carboxy-MoS_2_. **(c)** Zeta potential distribution curve of bulk MoS_2_ and single-layer carboxyl-MoS_2_ nanocomposites in water solution. **(d)** Nyquist plots of the impedance analysis at different chip interfaces. **(e)** CV curves at various scan rates of the Au/Cys/carboxyl-MoS_2_ sensing chip.

In this experiment, the successful modification of carboxyl-MoS_2_ nanocomposites was confirmed by transmission electron microscopy (TEM), Fourier transform infrared (FTIR), zeta potential, electrochemical impedance spectroscopy (EIS), and cyclic voltammetry (CV) analysis as shown in Figure 2. The TEM images clearly showed the surface structure of the prepared single-layer carboxyl-MoS_2_ nanocomposite (Figure 2a). Carboxyl led to hydrophilic functional groups on the MoS_2_ surface, which exhibited the structural characteristics of an organic chitosan matrix. The structure of these glycan matrices enhanced the surface area of –COOH and can enhance the formation of covalent bonds between proteins.

The FTIR spectrum of chloroacetic acid-modified single-layer MoS_2_ nanocomposite (Figure 2b) showed hydroxyl (–OH), carboxyl (O=C–O), and alkane (C–H) groups, as indicated by the characteristic peaks of C, O, and H vibrations. Compared to MoS_2_, the appearance of characteristic absorption peaks at 1290, 1440, 1594, and 1731 cm^–1^ in the spectrum of carboxyl-MoS_2_ corresponded to the C–O, –OH, C=C, and O=C–O stretching vibrations of –COOH, respectively. Among them, the absorption bands at 1380, 2848, and 2923 cm^–1^ in the spectrum corresponded to C–H bending vibrations. The absorption peak at around 3340 cm^–1^ was characterized as the O–H stretching vibration of –COOH. In addition, the absorption peak at 1060 cm^–1^ corresponded to the C–O stretching vibration for alcohol groups (Yang et al., 2016).

Zeta potential analysis (Nano ZS, Malvern Panalytical Ltd., United Kingdom) was used to investigate the carboxyl-functionalized MoS_2_ nanocomposite stability of dispersion. The results showed values of −32.5 mV for MoS_2_ and −51.4 mV for carboxyl-MoS_2_ nanocomposite. The formation of surface–COOH groups on the carboxyl-MoS_2_ nanocomposite and the increase in surface charge implied that the sulfide surface had successfully been modified by carboxyl as shown in Figure 2c. Therefore, the dispersion stability of modified carboxyl-MoS_2_ in water had a strong relationship with conductivity and zeta potential. Of note, the zeta potential values were consistent with previously reported results (Chou et al., 2013).

Figures 2d,e show the electrochemical measurements of EIS and CV analysis using a CHI model 604D workstation (CH Instruments, Austin, TX, United States) for the dielectric properties of carboxyl-MoS_2_. The measurement conditions were 0.1 M KCl solution containing 2 mM [Fe(CN)_6_]^4–/3–^ at pH 7.0, and the frequency range was between 0.1 and 10^5^ Hz with a sinusoidal wave of 5 mV. The three-electrode system included the modified Au/Cys/carboxyl-MoS_2_ chip as the working electrode, platinum (Pt) wire as the counter electrode, and silver-silver chloride (Ag/AgCl, KCl 3.0 mol/L) as the reference electrode.

Electrochemical impedance spectroscopy was used to obtain Nyquist plots to analyze differences in the activity among the interface impedance of bare Au, Au/Cys, Au/Cys/MoS_2_, and Au/Cys/carboxyl-MoS_2_ chips. The results revealed that the bare Au chip had an electron transfer resistance (*R*_*et*_) of 53.51 Ω. After incubation with the non-conductive linker Cys to capture probes on the bare Au chip, the *R*_*et*_ increased to about 200.4 Ω, indicating that the Cys film greatly limited electron transfer. Further immobilization of MoS_2_ showed a higher *R*_*et*_, and the Au/Cys/MoS_2_ chip had an *R*_*et*_ of 432.4 Ω, which was caused by the hindrance of electron transfer. In addition, immobilization of carboxyl-MoS_2_ resulted in an *R*_*et*_ of 275.7 Ω for the Au/Cys/carboxyl-MoS_2_ chip, which may be ascribed to the presence of carboxylate COO– groups on the chip surface. The carboxyl-modified MoS_2_ exhibited unique characteristics such as large surface area and good conductivity, which resulted in more ferro-ferricyanide participating in the reaction. Compared with MoS_2_, these results showed that the Au/Cys/carboxyl-MoS_2_ chip had low resistance due to rapid electron transfer at the carboxyl-modified surface. This may have been because carboxyl contributed to more conductive pathways for electron transfer, possibly due to the large surface area and compactness as well as a decrease in surface defects of carboxylate anions on the electrode surface (Bharath et al., 2015; Zheng et al., 2017). Surface modification of MoS_2_ by negatively charged carboxyl-groups to yield carboxyl-MoS_2_ could further improve aqueous dispersion, reduced damping and enhance biological affinity. These findings show that carboxyl-MoS_2_ could be used as a modifier to construct biosensors with improved plasmon damping and increased Interface electric field amplitude performance.

Figure 2e shows the CV curves which were obtained at potentials ranging from −0.4 to 0.7 V and scan rates from 15 to 150 mv/s for the carboxyl-MoS_2_ chip. The 150 mv/s scan rate showed peak potential separations (Δ*E*_*p*_ = 0.299 v) of 0.089 to 0.388 v of oxidation to reduction peak potential, respectively. Moreover, the scan rate of 15 mv/s showed peak potential separations (Δ*E*_*p*_ = 0.173 v) of 0.146–0.319 v. The ΔEp values did not change greatly with different scan rates, indicating that the carboxyl-MoS_2_ modification was stable and a good sensing nanocomposite. In addition, the cathodic and anodic peak current ratios (Ipc/Ipa) were 0.975 and 0.996 at scan rates of 15 mv/s and 150 mv/s, respectively, close to the theoretical value of 1 for a reversible reaction (Wu et al., 2016). A small ΔEp value indicated a fast electron transfer rate resulting in enhanced charge transfer kinetics (Nicholson, 1965) and sensitivity at the sensing chip of the carboxyl-MoS_2_ nanocomposite, indicating that carboxyl and MoS_2_ could be used as a modifier to construct biosensors with improved performance. Hence, the carboxyl-modified MoS_2_ exhibited a low R_*et*_ value, suggesting that carboxyl could be used as an optimized electrode for electrochemical analysis based on the obtained lower R_*et*_ value. Supplementary Figures S1 shows X-ray photoelectron spectroscopy (XPS) analyses of the Mo, S, C 1s, and O 1s characteristic peak for the MoS_2_ and carboxyl-MoS_2_ chips. Successfully modified carboxyl-MoS_2_ nanocomposites were observed, and the carboxyl bonds to the surface of the MoS_2_ film increased the carboxyl group content by 34.1%.

### Analysis of Coupling Effect of the SPR Mode in Carboxyl-MoS_2_ Films

**FIGURE 3:**
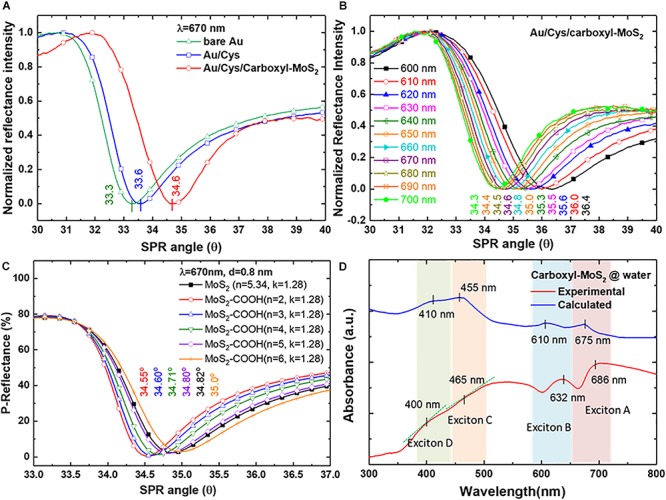
The experimentally measured reflectance SPR characteristic curves of **(A)** SPR angle for different chips at an incident wavelength of 670 nm, and **(B)** SPR curves measured for the Au/Cys/carboxyl-MoS_2_ chip at incident wavelengths from 660 to 700 nm. **(C)** Theoretical calculations for the Au/Cys/carboxyl-MoS_2_ chip. **(D)** Optical absorption spectra for carboxyl-MoS_2_ nanocomposite dispersion.

Studies of the chip structures and different medium layers to analyze SPR characteristic curves were used in SPR sensing films. In the SPR characteristic curves analysis, we used three different chips to measured resonance angle shifts at a fixed incident wavelength of 670 nm tested in an air medium environment as shown in Figure 3A. The SPR angle shifts were 33.3°, 33.5°, and 34.6° for bare Au, Au/Cys and Au/Cys/carboxyl-MoS_2_ chips, respectively. The results showed that more film layers led to larger SPR resonance angles. Figure 3B shows SPR angle shifts of the Au/Cys/carboxyl-MoS_2_ chip measured at different incident wavelengths, with corresponding incident wavelengths of 600, 610, 620, 630, 640, 650, 660, 670, 680, 690 and 700 nm at resonance angles of 36.4°, 36.0°, 35.6°, 35.5°, 35.3°, 35.0°, 34.8°, 34.6°, 34.5°, 34.4°, and 34.3°, respectively. The excitation conditions (the thickness of the sensing layer) are the same for all incident wavelengths. The results showed that larger incident wavelengths (700 nm) led to small SPR resonance angles. This may have been due to the increased number of surface oxygen and carboxyl groups in carboxyl-MoS_2_ (such as XPS O 1s and C 1_*S*_ as shown in Supplementary Figure S1), which resulted in a decrease in the refractive index of the film (Hinman et al., 2018).

Figure 3C shows theoretical calculation using Fresnel’s equations to evaluate the SPR reflectance curve of sensing chip structures and different medium interface parameters. The Kretschmann configuration of the SPR chip was considered to be a multilayer structure of BK7/Cr/Au/Cys/carboxyl-MoS_2_/air. SPR curves were calculated at incident angles (external angles) of 33°–37°. The layer-by-layer assembly of the SPR chip corresponded to the thicknesses (*d*) of the BK7 substrate, Cr film, Au film and Cys linker of 175 μm, 2 nm, 47 nm, and 0.5 nm, respectively. Previous studies have reported that the thickness of carboxyl-MoS_2_ is about 0.8 nm (Cheng Z. et al., 2015). Optical parameters of refractive index (*n*) and extinction coefficient (*k*) were evaluated using Fresnel’s multilayers model for the carboxyl-MoS_2_-based SPR chip. The *n* and *k* values at an incident wavelength (λ_*i*_) of 670 nm corresponding to the BK7, Cr, Au, and Cys films were BK7 = 1.514 + i0, Cr = 3.718 + i4.366, Au = 0.138 + i3.561, Cys = 1.45 + i0 (Rossi et al., 2003), and MoS_2_ = 5.34 + 1.28 (Beal and Huges, 1979), respectively. Moreover, we defined carboxyl-MoS_2_ and MoS_2_ to have the same extinction coefficient (*k*) of 1.28 to fit the calculations for the SPR angle (this assumed that the –COOH modification of MoS_2_ did not change the extinction coefficient due to non-metallic materials). The *n* and *k* values of these materials temperature was assumed to be 25°C to calculations for the SPR angle responses.

Therefore, we used three conditions (*d* = 0.8 nm, λ_*i*_ = 670 nm and *k* = 1.28) of carboxy-MoS_2_ for the reverse derivation of the SPR angle at different refractive index (*n*) constants. Figure 3C shows the results of calculations for a carboxyl-MoS_2_-based chip, with corresponding refractive index constants of 2, 3, 4, 5, and 6 at SPR angles (θ_*SP*_) of 34.55°, 34.60°, 34.71°, 34.80°, and 35.00°, respectively. The results showed that the SPR angle of the MoS_2_-based chip was 34.82°, and that the carboxyl-MoS_2_-based chip with *n* = 3 and *k* = 1.28 to fit the calculation had the same SPR angle (34.60°) as the measured results shown in Figure 3B at a wavelength of 670 nm. The results showed that the refractive indices of carboxyl-MoS_2_ in the larger-wavelength region were smaller than those in the short-wavelength region, which resulted in small shifts of the measured SPR angle (whereas a shorter incident wavelength will result in a larger SPR angle). This phenomenon is due to the refractive index (*n*) and extinction coefficient (*k*) of the material resulting in different SPP dispersion characteristics of the absorbed wave in different wavelength regions (Tanabe et al., 2017; Kuai et al., 2019).

Figure 3D shows the UV–visible absorption spectrum of measured and calculated values for carboxyl-MoS_2_ dispersion in water. The measured absorption band appearance was remarkable at two excitons of the A and B peaks for 686 and 632 nm, respectively. The short wavelength part (C and D peaks) of the spectrum split the bands into two peaks at 400 and 465 nm, which are known to arise from direct-gap optical transitions between a higher density of state regions of the MoS_2_ band structure in the K-point of the Brillouin zone (Fan et al., 2016; Jia et al., 2016).

Our experimental measurement results show that the absorption peaks of the spectra at 400 nm and 465 nm could detect slight changes in the slope. These absorption spectral characteristics of the carboxyl-MoS_2_ materials are similar to previous studies (Woodward et al., 2014; Jia et al., 2016). In Figure 3D, the synthesis of carboxyl-MoS_2_ nanocomposites process temperature was 100°C, and the material temperature of the calculation module was assumed to be 25°C. In addition, by comparing the experimental curve and the calculated curve, it can be seen in the experimental curve that the characteristic peaks of exciton A (686 nm), exciton B (632 nm), and exciton C (465 nm) regions were red shifted, while the characteristic peaks of exciton D (400 nm) were blue shifted. This is because the process temperature during the synthesis of carboxyl-MoS_2_ nanocomposites changes the refractive index of the MoS_2_ material, so the refractive index has a displacement effect on the absorption spectrum (Yalon et al., 2017). Therefore, it is correct and reasonable to find that the refractive index of carboxyl-MoS_2_ (*n* = 3) was smaller than that of MoS_2_ (*n* = 5.34) in the experimental reasoning calculation (Figure 3C). Moreover, it can be seen in Figure 3D that the lower refractive index after the modification of carboxyl-MoS_2_ caused exciton A, B, and C regions to be red shifted, but exciton D was shifted to a short wavelength. This suggests that the carboxyl-MoS_2_ films in our SPR chips were responsible for the increased value of absorbance, confirming absorption of light in the red-wavelength region corresponding to photon energies below the carboxyl-MoS_2_ bandgap. The results showed that the carboxyl-MoS_2_ optical absorption characteristics were the same as for typical MoS_2_ dispersions. Compared with the theoretical fit calculations (*n* = 3, *k* = 1.28) based on carboxyl-MoS_2_, the results showed that the absorption spectra of A (675 nm) and B (610 nm) excitons with C (455 nm) and D (410 nm) peaks had corresponding matching positions.

### Kinetic Analysis of Binding Interaction Between Carboxyl-MoS_2_ and Anti-CYFRA21-1 Protein

**FIGURE 4:**
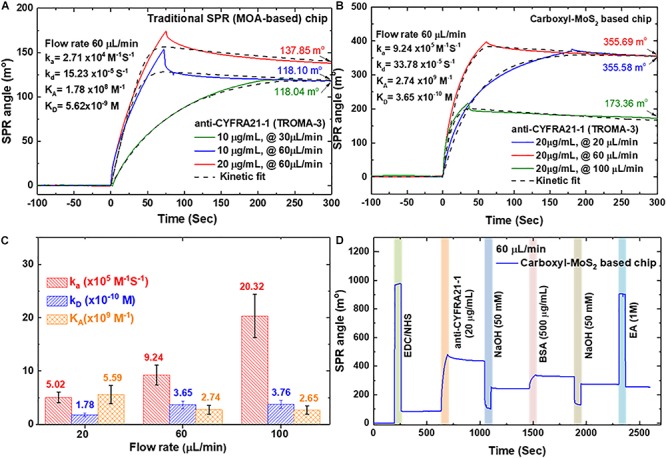
SPR kinetics analysis was obtained by using a different concentration and flow rate of anti-CYFRA21-1 immobilized on a sensing chip. **(A)** Traditional SPR chip. **(B)** Carboxyl-MoS_2_-based chip. **(C)** Binding analysis of different flow rates for the carboxyl-MoS_2_ based chip. **(D)** SPR sensorgram of the carboxyl-MoS_2_-based chip showing the success of the continuous immobilization process.

Studies of kinetic and dynamic interactions of protein adhesion under controlled conditions of both flow rate and chemical environment are particularly important. We used the functionalized interfaces of the carboxyl-MoS_2_ nanocomposites to capture and analyze affinity through binding to anti-CYFRA21-1 proteins. Figures 4A,B show the interaction analysis of a traditional SPR (MOA-based) chip and carboxyl-MoS_2_-based chip with different flow rates to evaluate the binding reactions of anti-CYFRA21-1 protein binding efficiency in real-time.

Figure 4A shows a traditional SPR chip (MOA-based on 47-nm thick Au film) in which antibodies at concentrations of 10 and 20 μg/mL were injected at different flow rates (30 and 60 μL/min) to observe binding and interaction reactions. At a flow rate of 60 μL/min, the SPR angle shifts with anti-CYFRA21-1 concentrations of 10 and 20 μg/mL were 118.10 and 137.85 millidegrees (m°), respectively. In addition, the SPR angle shift caused by the injection of a concentration of 10 μg/min at a flow rate of 10 μL/min was 118.04 m°, which was similar to the shift angle at an injection flow rate of 60 μL/min. The results showed that the angular shift caused by the traditional SPR (MOA-based) chip was similar at flow rates of 30 and 60 μL/min. We performed a 1:1 interaction of the SPR biosensor sensing surface with anti-CYFRA21-1 protein using the binding Langmuir model (Bumba et al., 2018). The kinetic analysis for the traditional SPR chip at a flow rate of 60 μL/min yielded an affinity binding constant (*K*_*A*_) of 1.78 × 10^8^ M^–1^, association rate constant (*k*_*a*_) of 2.71 × 10^4^ M^–1^S^–1^ and dissociation rate constant (*k*_*d*_) of 15.23 × 10^–5^ S^–1^.

Figure 4B shows that the shifts in the SPR angle for the carboxyl-MoS_2_-based chip with an anti-CYFRA21-1 concentration of 20 μg/mL at three different flow rates of 20, 60, and 100 μL/min at equilibrium were 355.58, 355.69, and 173.36 m°, respectively. The results showed that the adhesion of anti-CYFRA21-1 protein on the carboxyl-MoS_2_-based chip at flow rates of 20 and 60 μL/min reached maximum angle shifts. The kinetic binding analysis showed an enhanced SPR angle response for each flow rate of the carboxyl-MoS_2_-based chip, while low binding was recorded for the traditional SPR chip. This was similar to the traditional SPR chip, where anti-CYFRA21-1 protein adhesion increased the SPR angle shift at flow rate of 60 μL/min, where a maximum SPR angle shift was reached. A higher flow rate of 100 μL/min resulted in a gradual decrease in the SPR angle signal. Figure 4C shows the kinetic analysis for the carboxyl-MoS_2_-based chip, in which the affinity binding *K*_*A*_ values at 20, 60, and 100 μL/min were 5.59 × 10^9^, 2.74 × 10^9^, and 2.65 × 10^9^ M^–1^, respectively. A flow rate of 60 μL/min resulted in a *k*_*a*_ value of 9.24 × 10^5^ M^–1^S^–1^, *k*_*d*_ of 33.78 × 10^–5^ S^–1^, affinity binding *K*_*A*_ of 2.74 × 10^9^ M^–1^, and dissociation phase *K*_*D*_ of 3.65 × 10^–10^ M. Compared to the *K*_*D*_, in all flow rate was approximate similar because the dissociation phase does not depend on flow rates. This means that the experiments of mass transport are not obvious.

Compared to the traditional SPR chip, the carboxyl-MoS_2_-based chip had significantly improved binding affinity and sensitivity at a flow rate of 60 μL/min, with a 2.6-fold increase in SPR angle and 15-fold increase in affinity binding *K*_*A*_.

Figure 4D shows a series of consecutive real-time sensorgrams of the carboxyl-MoS_2_-based chip for the immobilization process in 1× PBS buffer and injections of all samples at a flow rate of 60 μL/min and volume of 200 μL. We first injected EDC/NHS mixture for 200 s to activate the carboxyl-MoS_2_-based chip surface. Immobilization of the anti-CYFRA21-1 protein was then performed by –COOH coupling on the chip surface. Anti-CYFRA21-1 protein diluted to 20 μg/μL in 1× PBS buffer was then injected for 630 s, and unbound and non-specifically adsorbed anti-CYFRA21-1 protein was washed away through injections of 50 mM NaOH at pH 12.0. We then injected a high concentration of BSA protein (500 μg/mL) for 1460 s to block the remaining –COOH bonds to reduce non-specific electrostatic adsorption, followed by a further injection of 50 mM NaOH for 1880s to wash away unbound and dissociate non-specifically adsorbed BSA proteins. Finally, the unreacted –COOH groups on the surface were deactivated using an injection of small-molecule 1 M EA solution for 2300s.

### Analysis of the Detection of CYFRA21-1 Protein Using the Carboxyl-MoS_2_ Based Chip in Spiked Human Clinical Serum Samples

**FIGURE 5:**
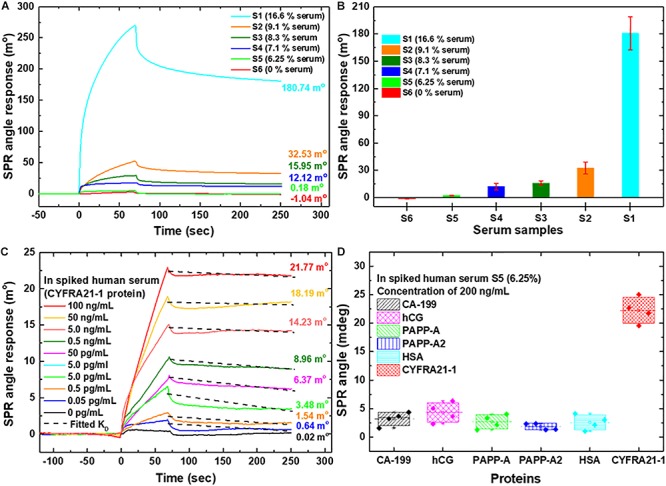
SPR sensorgram curves were analyzed for the CYFRA21-1 protein in spiked human serum samples using the carboxyl-MoS_2_-based chip. **(A)** Different serum concentrations were used to assess interference analysis during the test. **(B)** Comparisons of different serum ratios at SPR biosensing for interference analysis. **(C)** SPR curves for the quantitative detection of CYFRA21-1 protein using the S5 buffer spiked with different dilutions of CYFRA21-1 proteins. **(D)** The specificity of the biosensor tested the SPR responses to non-specific interfering biomarkers. The SPR responses to CA-199, hCG, PAPP-A, PAPP-A2, HSA, and CYFRA21-1 proteins mixed with S5 (6.25% serum) at a ratio of 1:1 in a total volume of 200 μL buffer were investigated.

In the assay for CYFRA21-1 in spiked clinical serum, the presence of CYFRA21-1 antigen was defined as positive responses on the carboxyl-MoS_2_-based SPR chip. The dynamic processes of the assay were controlled by anti-CYFRA21-1 and CYFRA21-1 binding affinity and specificity interactions. Figure 5A shows the SRB buffer with different spiked ratios of serum samples to assess possible interference caused by serum.

In order to identify the optimum conditions under which a carboxyl-MoS_2_-based SPR chip showed non-specific interference, we set six different ratios of conditions as 0.5 mL PBS_(B__*TN*__)_ buffer mixed with 0.1 mL serum for S1 (16.6% serum), 1.0 mL PBS_(BTN)_ buffer mixed with 0.1 mL serum for S2 (9.1% serum), 1.0 mL PBS_(__*BTN*__)_ buffer mixed with 0.1 mL serum and 0.1 mL 50 mM NaCl for S3 (8.3% serum), 1.0 mL PBS_(BTN)_ buffer mixed with 0.1 mL serum and 0.3 mL 50 mM NaCl for S4 (7.1% serum), 1.0 mL PBS_(BTN)_ buffer mixed with 0.1 mL serum and 0.5 mL 50 mM NaCl for S5 (6.25% serum), and PBS_(BTN)_ buffer for S6 (0% serum), respectively.

We observed that the SPR angle shifts of the different ratios of the spiked serum samples (S1, S2, S3, S4, S5, and S6) were 180.74, 32.53, 15.95, 12.12, 0.18, and −1.04 m°, respectively. The experimental results showed that the shift in the SPR sensorgram reaction angle of S1 was the largest. The interference indicating the adsorption of non-specific molecules with the S1 buffer was very serious. Increasing the serum dilution resulted in a decrease in the SPR angular displacement (S2 buffer), and could effectively reduce the interference caused by serum. In addition, dilutions of the serum samples (S3, S4, S5) and the addition of NaCl could more effectively reduce the interference of non-specific molecules. The SPR sensorgrams of the six different conditions with the experiments repeated three times are shown in Figure 5B. S5 (6.25% serum) buffer was selected as the optimum conditions for the detection experiments. We then analyzed the detection of CYFRA21-1 protein in spiked human serum S5 (6.25% serum) buffer with the carboxyl-MoS_2_-based SPR chip, and further examined the non-specific binding of the chip by subjecting it to serum samples spiked with CYFRA21-1 protein at different concentrations.

We used the initial samples mixed with S5 (6.25% serum) buffer at a ratio of 1:1 in a total volume of 200 μL. This resulted in 3.125% serum with CYFRA21-1 protein concentrations of 0 pg/mL, 0.05 pg/mL, 0.5 pg/mL, 5 pg/mL, 50 pg/mL, 0.5 ng/mL, 5 ng/mL, 50 ng/mL, and 100 ng/mL. CYFRA21-1 protein samples were injected at a flow rate of 60 μL/min for 0-70 s to allow the analyte of CYFRA21-1 in the sample to interact and bind to the surface of anti-CYFRA21-1 protein. We then injected S5 buffer for 70 s to rinse the surface. The SPR sensorgrams showed that the CYFRA21-1 interactions with anti-CYFRA21-1 protein gradually stabilized (85–250 s) at an equilibrium time of 250 s. The *K*_*D*_ was similar for all concentrations because the dissociation rate does not depend on antigen concentration. The sensorgrams were obtained with CYFRA21-1 concentrations of 0 pg/mL, 0.05 pg/mL, 0.5 pg/mL, 5.0 pg/mL, 50 pg/mL, 0.5 ng/mL, 5.0 ng/mL, 50 ng/mL, and 100 ng/mL, and the SPR angle shift responses were 0.02, 0.64, 1.54, 3.48, 6.37, 8.96, 14.23, 18.19, and 21.77 m°, respectively, as shown in Figure 5C. The experimental results showed that the carboxyl-MoS_2_-based SPR chip had excellent detection performance, and that it could be used in spiked human serum to detect CYFRA21-1 concentrations with a measured LOD of 0.05 pg/mL. Furthermore, Figure 5C shows that the kinetic binding efficiency of the dissociation constant (*K*_*D*_) ranged from 252.6 × 10^–9^ to 980.3 × 10^–9^ M in the different CYFRA21-1 concentrations.

To evaluate the specificity tests in the carboxyl-MoS_2_-based SPR biosensor with various biomarkers, we tested non-specific interference with molecular interaction assays on an anti-CYFRA21-1 probe and six different biomarkers, as shown in Figure 5D. The SPR angle responses of the six different biomarkers were measured four times. The measured average SPR angle responses for each biomarker mixed with S5 (6.25% serum) at a ratio of 1:1 in a total volume of 200 μL for CA-199, hCG, PAPP-A, PAPP-A2, HSA, and CYFRA21-1 were 3.65, 5.02, 4.02, 2.35, 3.02, and 21.77 m°, respectively, as shown in Figure 5D. Moreover, the carboxyl-MoS_2_-based SPR biosensor in non-specific interference tests showed good reproducibility [relative standard deviation (RSD) of 5.26% (four replicates, *n* = 4)]. The experimental results showed that in a complex serum environment with many ions and sums, the biosensor did not react with other interfering biomarkers. The results showed that the SPR biosensor based on carboxy-MoS_2_ had high specificity.

**FIGURE 6:**
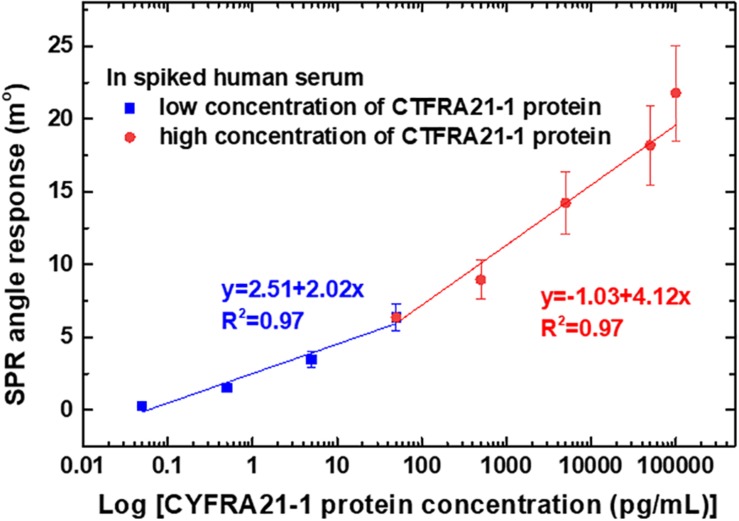
Calibration curves of average SPR detection with various CYFRA21-1 protein concentrations ranging from 0.05 pg/mL to 100 ng/mL. The error bars represent the SD of three replicates.

The calibration curves obtained for different dilutions of CYFRA21-1 protein concentrations in spiked human serum for linear regression with error bars representing the standard deviation (SD) of three replicates (*n* = 3) are shown in Figure 6. The SPR angle responses of the different concentrations of CYFRA21-1 protein were measured three times. The calibration curves were comprised of two sections with different slopes according to a low (0.05–50 pg/mL) and high (50 pg/mL–100 ng/mL) concentration dynamic range. The results showed two ranges in which the log linear regression had higher linearity. The low concentration linear range had a regression equation of *y* = 2.51 + 2.02*x* and a correlation coefficient (*R*^2^) of 0.97, while the high concentration linear range had a regression equation of *y* = 1.03 + 4.12*x* and *R*^2^ of 0.97, where *x* is the logarithm of CYFRA21-1 protein concentration [log (pg/mL)], and *y* is the SPR angle (m°).

The average SPR angle displacement signal-to-noise ratio (SNR) was 33.4 dB for high concentrations (50 pg/mL, 0.5 ng/mL, 5 ng/mL, 50 ng/mL, and 100 ng/mL) of CYFRA21-1 protein. In addition, the average SPR angular displacement SNRs were 21.5 dB for low concentrations (0.05, 0.5, and 5.0 pg/mL) of CYFRA21-1 protein. The SPR angle displacement of carboxyl-MoS_2_-based SPR chip has a smaller baseline noise <0.01 m° root mean square. Moreover, the carboxyl-MoS_2_-based SPR biosensor showed good reproducibility of 95%. These experiments also demonstrated a LOD of the carboxyl-MoS_2_-based SPR chip of 0.05 pg/mL, which we determined from the low noise and the relationship between the relative SPR angle response and CYFRA21-1 concentration. This statistical result showed that the carboxyl-MoS_2_-based SPR chip could accurately and stably detect CYFRA21-1 protein in spiked human serum.

In addition, we also analyzed and verified the reliability of the detection of the carboxyl-MoS_2_-based SPR chip in spiked human serum. The recovery tests were performed using different spiked human serum buffers (S4, S5, and S6) to detect CYFRA21-1 protein. Known quantities of CYFRA21-1 protein of 5.0 pg/mL, 50 pg/mL, 0.5 ng/mL, and 5.0 ng/mL were then used in the recovery test experiments of four replicates. The results for different diluted spiked human serum samples of the recovery rates of S4, S5, and S6 buffers were 70–115, 85–104, and 90–103%, respectively (Table 1). In the S5 buffer, the recovery results were 85–102, 87–104, 88–100, and 90–102% for 5.0 pg/mL, 50 pg/mL, 0.5 ng/mL, and 5.0 ng/mL concentrations of CYFRA21-1, respectively, with a RSD below 8.1%. In the S5 buffer, the average recovery rate was close to 94.5%. These results showed the excellent binding affinity, high sensitivity, low detection limit, good stability and specificity. In summary, the carboxyl-MoS_2_-based SPR chip could reliably detect CYFRA21-1 protein in spiked human serum.

**TABLE 1:** Determination and recovery rates to detect CYFRA21-1 protein at different concentrations in spiked human serum using the carboxyl-MoS_2_-based SPR chip.

**Concentration**	**S4 buffer (7.1% serum)**		**S5 buffer (6.25 % serum)**		**S6 buffer (0 % serum)**	
	**Recovery (%)**	**RSD (%)**	**Recovery (%)**	**RSD (%)**	**Recovery (%)**	**RSD (%)**
5.0pg/mL	70-115	**19.9**	85-102	8.1	92-103	5.1
50 pg/mL	76-107	14.1	87-104	7.7	95-103	4.2
0.5 ng/mL	78-110	15.3	88-100	5.4	92-102	4.4
5.0ng/mL	81-112	13.3	90-102	5.5	90-100	4.5

## Conclusion

The proposed novel carboxyl-functionalized MoS_2_ nanocomposite in this study could enhance SPR sensing performance and may open new perspectives for applications in detecting cancer biomarkers in the field of SPR-based immunosensors platforms. This study successfully confirmed the –COOH-modified effects of increased surface chargeability, improved conductivity, and high-speed electron transfer rate with carboxyl-MoS_2_. Compared with MoS_2_, the results showed the formation of shell-like glycan matrices, increased –COOH content of 34.1%, zeta-potential and electronegativity of −51.4 mV, low resistance of 275.7 Ω, and low refractive index obtained by a fitting calculation of 3. Surface modification of the carboxyl-MoS_2_ nanocomposite was an effective way to increase the interaction binding and affinity of carboxyl-MoS_2_ and proteins. A significant decrease in fouling and a smaller SPR angular shift were achieved when the serum was diluted to 6.25% (S5) in SRB buffer, and this could effectively reduce interference. In this study, we successfully detected a lung cancer biomarker in CYFRA21-1-spiked serum sample in the presence of serum, and established distinct multiplex assays with minimum cross-reaction of anti-CYFRA21-1 proteins. We also observed that the SPR response resulting from the binding of carboxyl-MoS_2_ and anti-CYFRA21-1 gradually increased with an increase in CYFRA21-1 concentration from 0.05 pg/mL to 100 ng/mL. The resulting LOD for the carboxyl-MoS_2_-based chip was 0.05 pg/mL, which caused an angle shift of 0.64 m° in spiked human 3.125% serum. Further research on the application and miniaturization of carboxyl-MoS_2_-based SPR chips may lead to their use in routine clinical trial analysis and point-of-care testing (POCT) diagnostics.

## Data Availability Statement

The datasets generated for this study are available on request to the corresponding author.

## Ethics Statement

The studies involving human participants were reviewed and approved by The authors would like to thank the Mackay Hospital, Taipei, Taiwan, this work was approved by the Institutional Review Board (IRB) of Mackay Hospital for Human Clinical Trials (Permit Numbers: 15MMHIS020, 15MMHIS115 and 17MMHIS185). The patients/participants provided their written informed consent to participate in this study.

## Author Contributions

H-TY and N-FC performed the device fabrication and collected the experimental data. N-FC provided guidance for the experimental setups, performed data analysis, wrote the manuscript, and oversaw the project and performed the overall editing of the manuscript.

## Conflict of Interest

The authors declare that the research was conducted in the absence of any commercial or financial relationships that could be construed as a potential conflict of interest.
