# Supplementary material for: High-Sensitivity Detection of the Lung Cancer Biomarker CYFRA21-1 in Serum Samples Using a Carboxyl-MoS2 Functional Film for SPR-Based Immunosensors
Source: Front Bioeng Biotechnol. 2020 Mar 26;8:234. doi: 10.3389/fbioe.2020.00234 (PMC7113369; doi:10.3389/fbioe.2020.00234)
Supplement: Supplementary file 1 [file Data_Sheet_1.DOCX]

**Supplementary data**

# High-sensitivity detection of the lung cancer biomarker CYFRA21-1 in serum samples using a carboxyl-MoS_2_ functional layer for SPR-based immunosensors

Nan-Fu Chiu^1,2^*, Hao-Tang Yang^1^

^1^Laboratory of Nano-photonics and Biosensors, Institute of Electro-Optical Engineering, National Taiwan Normal University, Taiwan

^2^Department of Life Science, National Taiwan Normal University, Taiwan

**Corresponding author:**

^1,2*^TEL: +886-77346722 and Fax: +886-2-86631954 and E-mail: [nfchiu@ntnu.edu.tw](mailto:nfchiu@ntnu.edu.tw)

No. 88, Sec. 4, Ting-Chou Road, Taipei 11677, Taiwan

**S1. Preparation of traditional SPR immunosensors:**

For the preparation of the traditional SPR chip, we used the same chip substrate conditions with a BK7/Cr/Au structure. We then used the linker 8-mercaptooctanoic acid 95% (MOA; Sigma-Aldrich Co. LLC., St. Louis, MO, USA) to modify the thiol bond on the Au surface. MOA is a carboxylic acid-terminated thiol molecule that can form a chemical covalent bond with an Au surface resulting in a bare -COOH bond. We used 0.4 mM EDC/0.1 mM NHS at a 1:1 ratio to activate the carboxyl groups on the MOA linker, which could covalently immobilize the -NH_2_ terminus of 20 μg/mL anti-CYFRA21-1. We blocked the -COOH terminus of the unbound anti-CYFRA21-1 protein remaining on the carboxyl-MoS_2_ surface using 500 μg/mL BSA, and deactivated the remaining minority -COOH terminus with EA to prevent non-specific binding.

**S2. Analysis and verification of carboxyl-MoS_2_ nanocomposite in XPS spectra:**

In order to analyze the chemical modification of MoS_2_ by carboxyl, we used X-ray photoelectron spectroscopy (XPS) analysis to evaluate the surface composition elements and valence states. The representative XPS survey scan spectra of MoS_2_ and carboxyl-MoS_2_ nanocomposites are shown in Figures S1(a) and S1(b), respectively. The XPS spectra indicated the existence of Mo, S, carbon C, and O element concentrations corresponding to 37.6%, 25.1%, 24.3% and 13.0% for MoS_2_ and 37.0%, 21.5%, 22.8% and 18.7% for carboxyl-MoS_2_ nanocomposites, respectively. Compared to MoS_2_, carboxyl-MoS_2_ showed a relative increase in C and O elements due to the chloroacetic acid modification of the MoS_2_ nanocomposite. We observed that the relative O/C intensity ratios (I_O_/I_C_) were 0.535 and 0.823 for the MoS_2_ and carboxyl-MoS_2_ chips, respectively. In surface chemical bonding states, a high resolution XPS spectrum was used to analyze the binding energies of the carboxyl-MoS_2_ chip. Figure S1(c) shows XPS spectra analysis of Mo 3d and S 2s binding energies. The two peaks of Mo 3d were attributed to the doublet seen at 229.63 eV and 232.78 eV corresponding to Mo^4+^3d_5/2_ and Mo^4+^3d_3/2_, respectively. Moreover, the S 2s binding energy exhibited a peak at 226.83 eV and a small peak of Mo^6+^3d_3/2_ binding energy at 235.98 eV (Ho et al., 2015; Ahn et al., 2015). As shown in Figure S1(d), the S 2p two binding energy peaks exhibited 2p_1/2_ and 2p_3/2_ doublets at 163.58 and 162.38 eV, respectively. In the S 2p spectrum of the Au/Carboxyl-MoS_2_ chip (Figure S1(d)), we observed a significantly narrower band of S 2p doublet compared to the previous study of MoS_2_ (0001) basal plane surface (Durbin et al., 1992; Bruix et al., 2015). This may indicate that the reaction of S was affected by the chemical element environment of Au and -COOH.

Figure S1(e) shows C 1s peaks of carboxyl-MoS_2_ at 284.7 and 289.1 eV resulting from C–C (65.9%) and O–C=O (34.1%) orbitals, respectively. The efficiency of the modified carboxyl-MoS_2_ showed a higher -COOH group content of 34.1%, mainly due to chlorine atoms occupying the sulfur vacancies from 25.1% (Figure S1(a), MoS_2_) to 21.5% (Figure S1(b), carboxyl-MoS_2_), allowing for the formation of a strong bonding effect. Figure S1(f) shows O 1s peaks at 531.7, 532.7 and 533.8 eV, corresponding to the C=O, C–O and O–C=O orbitals of carboxyl-MoS_2_, respectively. Calculations of the atomic concentrations showed significant percentages of C=O, C–O and O–C=O orbitals on MoS_2_-modified carboxyl film of 16.4, 56.1 and 27.5%, respectively. The XPS results of C 1s and O 1s peaks clearly indicated large increases in element concentrations on the surface of the O–C=O groups.


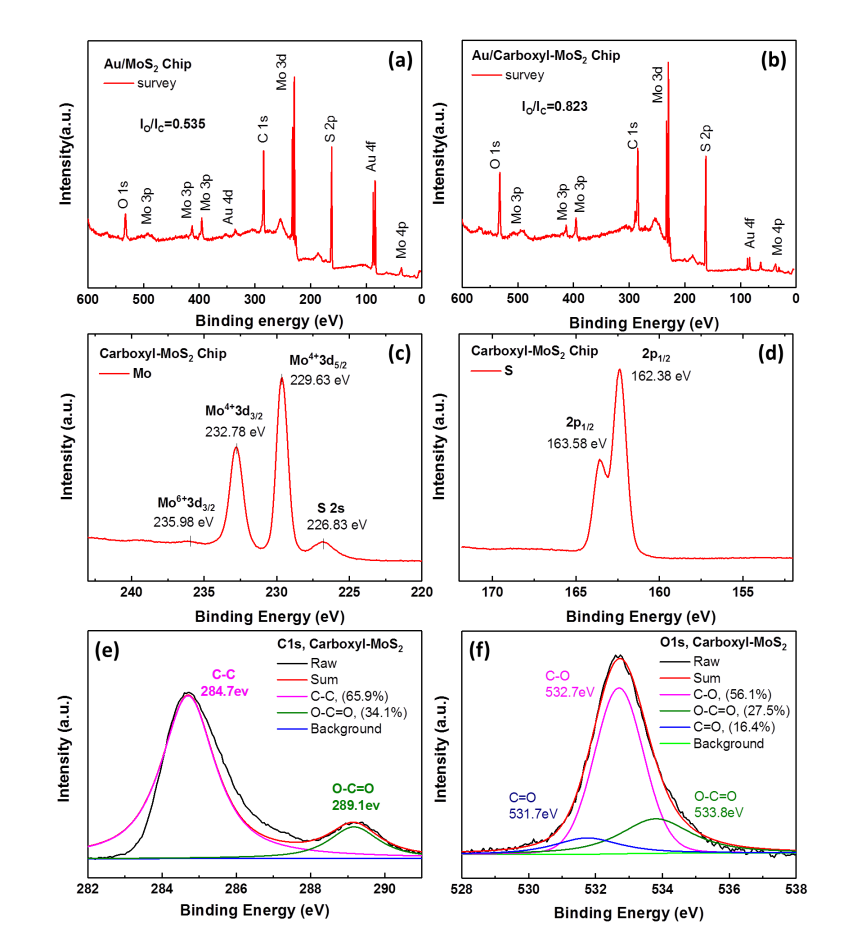


Figure S1. Survey XPS spectra of (a) MoS_2_ and (b) carboxyl-MoS_2_ nanocomposites with different mass ratios. High resolution XPS spectra of (c) Mo, (d) S, (e) C 1s, and (f) O 1s orbitals for the carboxyl-MoS_2_ chip.

**References**

Ahn, C., Lee, J., Kim, H.-U., Bark, H., Jeon, M., Ryu, G. H.,  Lee, Z., Yeom, G. Y., Kim, K., Jung, J., Kim, Y., Lee, C., and Kim, T. (2015). Low-temperature synthesis of large-scale molybdenum disulfide thin films directly on a plastic substrate using plasma-enhanced chemical vapor deposition. *Adv. Mater*. **27,** 5223–5229. doi:10.1002/adma.201501678

Bruix, A., Füchtbauer, H.G., Tuxen, A. K., Walton, A. S., Andersen, M., Porsgaard, S., Besenbacher, F., Hammer, B., and Lauritsen, J.V. (2015). In situ detection of active edge sites in single-layer MoS_2_ Catalysts. *ACS Nano* **9,** 9322–9330. doi:10.1021/acsnano.5b03199

Durbin, T. D., Lince, J. R., and Yarmoff, J. A. (1992). Chemical interaction of thin Cr films with the MoS2(0001) surface studied by x‐ray photoelectron spectroscopy and scanning Auger microscopy. *J. Vac. Sci. Technol. A* **10,** 2529–2534. doi:10.1116/1.578093

Ho, Y.-T., Ma, C.-H., Luong, T.-T., Wei, L.-L., Yen, T.-C., Hsu, W.-T., Chang, W.-H., Chu, Y.-C., Tu, Y.-Y., Pande, K.P., and Chang, E. Y. (2015). Layered MoS_2_ grown on *c*‐sapphire by pulsed laser deposition. *Phys. Status Solidi – Rapid Res. Lett*. **9,** 187–191. doi:10.1002/pssr.201409561
